# Measurements of the dose delivered during CT exams using AAPM Task Group Report No. 111

**DOI:** 10.1120/jacmp.v13i6.3934

**Published:** 2012-11-08

**Authors:** C. Descamps, M. Gonzalez, E. Garrigo, A. Germanier, D. Venencia

**Affiliations:** ^1^ Instituto de Radioterapia – Fundación Marie Curie Córdoba Argentina; ^2^ CEPROCOR Córdoba Argentina

**Keywords:** computed tomography, CT radiation dose, CTDI, quality assurance

## Abstract

The computed tomography dose index (CTDI) measured with a 10 cm long pencil ionization chamber placed in a 14 cm long PMMA phantom is typically used to evaluate the doses delivered during CT procedure. For the new generation of CT scanners, the efficiency of this methodology is low because it excludes the contribution of radiation scattered beyond the 100 mm range of integration along z. The AAPM TG111 Report proposes a new measurement modality using a small volume ionization chamber positioned in a phantom long enough to establish dose equilibrium at the location of the chamber. In this work, the AAPM report was implemented. The minimum scanning length needed to obtain cumulative dose equilibrium was evaluated. The equilibrium dose was determined and compared to CTDI values informed by the CT scanner, and the dose values were confirmed with TLD measurements. The difference between doses measured with TLD and with the ionization chamber (IC) was below 1% and the repeatability of the measurements' setup was 0.4%. The measurements showed that the scanning lengths needed to reach the cumulated dose equilibrium were 450 mm and 380 mm for the central and peripheral axes, respectively, which justifies the phantom length. For the studied clinical protocols, the doses measured were about 30% higher than those informed by the CT scanner. For the new generation of CT systems with wider longitudinal detector size or cone‐beam technology, the current CTDI measurements may no longer be adequate, and the informed CTDI tends to undervalue the dose delivered. It is therefore important to evaluate CT radiation doses following the AAPM TG111 methodology.

PACS number: 87.57.qp, 87.53.Bn

## I. INTRODUCTION

Computed tomography (CT) is one of the most frequently used diagnostic imaging methods. Recent overdose accidents^(1)^ led to new interest in evaluating the dose delivered during CT exams.^(2)^ The current paradigm used to evaluate these doses is the computed tomography dose index (CTDI), which represents the absorbed dose along the longitudinal axis (z‐axis) of the CT scanner measured during a single rotation of the X‐ray source. It is commonly measured with a 100 mm long pencil ionization chamber $\left({\text{CTDI}}_{100}\right)$ placed in a 14 cm long and 16 cm or 32 cm diameter cylindrical PMMA phantom representing adult head and body, respectively.^(3)^

With the new generation of CT scanners, advanced technologies with helical scanning mode or cone‐beam irradiation geometries appeared; therefore, the CTDI dose measurement methods were not suitable anymore.^4^, ^5^, ^6^, ^7^ Moreover, because of the increase of the detection system size along the z‐axis, the CT beams became larger and the use of ${\text{CTDI}}_{100}$ was not appropriate. This index excludes the contribution of radiation scattered beyond the 100 mm range of integration along the z‐axis and, in the case of larger cone‐beam geometry, the pencil chamber is too short to measure all of the primary radiation.

New measurement methods are described in the AAPM Task Group Report No. 111^(8)^ (AAPM TG111). The use of a small volume ionization chamber positioned in a phantom long enough to establish dose equilibrium at the location of the chamber is proposed. In this case, it is not the primary radiation that has to be evaluated, rather the dose at equilibrium. The methods and equations described in this report can be used for axial or helical scanning modes, for cone‐ or fan‐beam geometries, with or without table translation.

The aim of the present work was to follow the AAPM TG111 guidelines in order to evaluate the doses delivered during commonly used CT exam protocols, and to compare them with the doses informed by the CT scanner at the end of each scanning. The effective dose corresponding for each protocol was compared with international references. In order to complete the acceptance tests, free‐in‐air equilibrium dose was reported.

## II. MATERIALS AND METHODS

A Siemens, SOMATOM Spirit Power two‐slice CT scanner (Siemens AG, Munich, Germany) was used for these measurements. A PTW Farmer‐type chamber ($0.6\;{\text{cm}}^{3}$) connected to a PTW UNIDOS E electrometer (PTW, Freiburg, Germany) was calibrated by a Secondary Standard Dosimetry Laboratory (SSDL) for beam quality ranges associated with those of CT scanner spectra. The stem effect was evaluated for the entire setup (chamber, cable, and electrometer).

**Figure 1:**
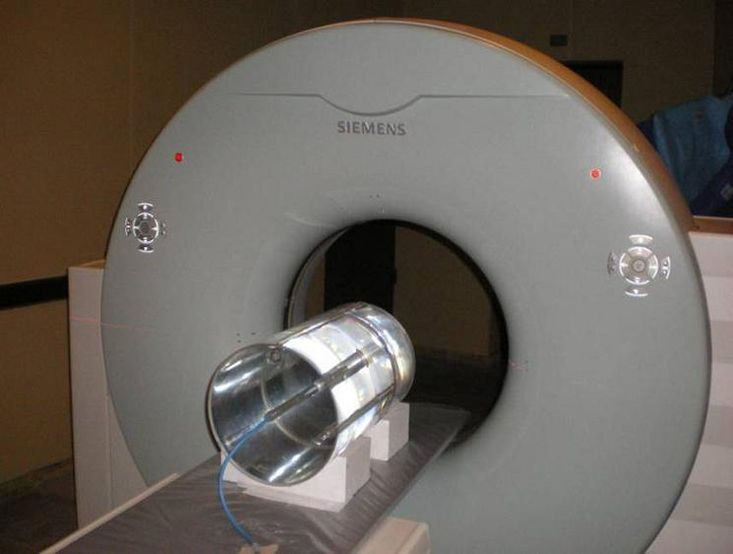
Water phantom with the ionization chamber placed in the central insert.

A 30 cm diameter, 50 cm long water phantom was designed to allow the chamber position at the center (Fig. 1) or at the peripheral axis. This phantom size was chosen in order to represent the attenuation and absorption characteristics of the average size adult body. The phantom was made in two sections for easy setup, with marks for repositioning on the CT table. The phantom material was taken into account for the measurements according to the IAEA Protocol TRS 277.^(9)^ A ratio of the spectrally averaged mass energy absorption coefficient of the phantom material (water) to that of air of 1.06 was used.^(10)^

**Figure 2:**
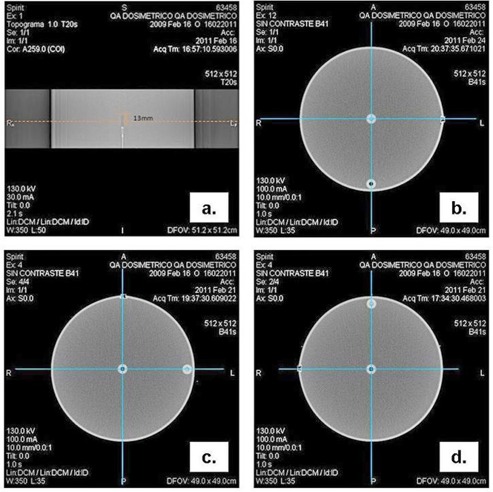
a) Positioning of the charge‐collection volume chamber with the CT z=0 plane; b), c) and d) Alignment of the phantom center with the CT central axis (image center) and verification of the position of the peripheral inserts.

Previous measurements were made in order to verify phantom and chamber correct positioning and dose repeatability. The water phantom central axis was aligned with the CT rotation axis, and the ionization chamber was placed in the phantom in order to center the charge collection volume with the plane z=0 of the CT scanner (Fig. 2). The electrometer was used in the control area, outside the scan room, and was connected to the ionization chamber with a special cable in order to reduce the extra chamber currents. The stem effect or noise signal of the system and the dose repeatability were evaluated.

The doses delivered during axial scanning acquisition with adjacent tomographic sections and helical scanning mode with a pitch factor of 1 were compared.

A “reference” set of operation was chosen following the AAPM TG111 recommendations as the most frequently used clinical protocol. For this work, the reference CT conditions were: axial‐scanning mode, adjacent slices, ${\text{kV}}_{\text{pref}}=130\;\text{kVp}$, 100 mA, 1 s per tube rotation, n=2‐slices of T=tomographic sections nominal width=5 mm, nT=total nominal width along the axis of rotation associated with the group of sections simultaneously acquired=10 mm.

**Figure 3:**
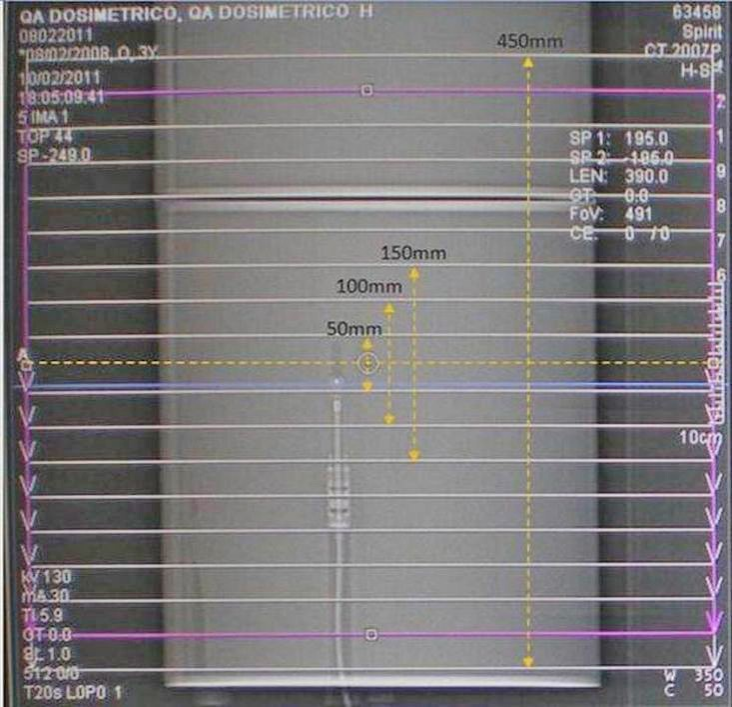
Scanning lengths realized to determine the cumulative central dose.

The minimum scanning length $L_{\text{eq}}$ needed to obtain the equilibrium cumulative dose $D_{\text{eq}}$ was evaluated. The cumulative central dose, $D_{L}$ (z=0)was measured with ionization chamber for a set of scanning lengths L ranging from 50 mm to the phantom length minus nT( Fig. 3). For each value of scanning lengths, $D_{L}(z=0)$ was characterized as the product of a function

“approach‐to‐equilibrium” h(L) (Eq. (1)) and the equilibrium dose D. The a parameter is related to the scatter‐to‐primary ratio (SPR) along the phantom axis.

(1^8^) $$h(L)=1-\alpha \exp(-4L/L_{eq})$$

The equilibrium dose was determined for the central and peripheral axes. The planar average equilibrium dose was calculated with the assumption that $D_{\text{eq}}$ is proportional to $r^{2}$ ^8^, ^11^ (Eq. (2)) and compared to CTDI values informed by the CT scanner.

(2^8^) $$D_{eq}=\frac{1}{2}D_{eq,central}+\frac{1}{2}D_{eq,peripheral}$$

The total energy absorbed in the phantom or integral dose $E_{\text{tot}}$ is a parameter used to assess the patient risk and was evaluated following Eq. (3) with $\rho =1190\;\text{kg}/m^{3}$, π=3.14, R=0.150 m, L=0.500 m.

(3^8^) $$E_{tot}=\rho \pi R^{2}LD_{eq}$$

The planar average equilibrium dose and the total energy absorbed were measured for frequently clinically used protocols in our radiotherapy institute. The set of parameters of each protocol is detailed in Table 1. The protocol 1 is used daily for chest 3D radiotherapy simulation treatment and protocol 2 for IMRT prostate simulation treatment. The third one is used for abdomen or metastasis treatment planning process when precise positioning system is used during the treatment. Protocol 4 was chosen to evaluate the influence of the pitch factor.

**Table 1:** Parameters of the clinical protocols used.

*PROTOCOLS*	*PROTOCOL 1*	*PROTOCOL 2*	*PROTOCOL 3*	*PROTOCOL 4*
Scanning mode	Helical	Axial	Axial	Helical
kVp	130	130	130	130
mA	100	100	100	100
n	2	2	2	2
T	5	1.5	2.5	2.5
"Pitch factor" =b/nT	1	1	1	1.5
Time per tube rotation	1s	1s	1s	1s

Notes: n=slice number, T=slice width, nT=total width tomographic sections, b=table increment in a sequence of axial scanning or continuous table advance per rotation during helical scanning.

**Figure 4:**
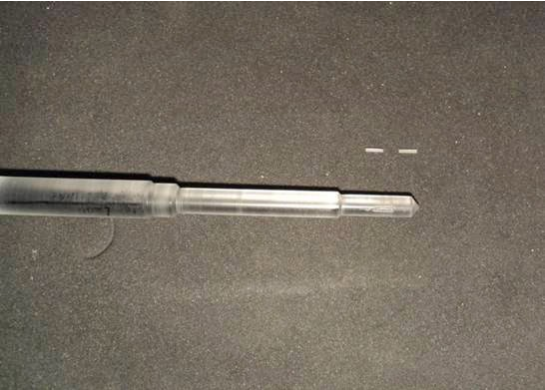
TLD rod and insert to realize measurements in the water phantom.

In order to validate the measurement set, doses were compared with TLD measurements. ${\text{TLD}}_{100}$ (rods of LiF:Mg,Ti, Harshaw) were used (Fig. 4). The TLDs were calibrated with a POLYMOBIL Plus X‐ray tube (Siemens AG, Munich, Germany). The quality of the X‐ray tube used to calibrate the TLD was HVL=0.23 mm of Cu. Using a Farmer‐type ionization chamber (calibration factor: $N_{k}=47.90\;\text{mGy}/\text{nC}$) and following the IAEA TRS‐277,^(9)^ the energy absorption coefficient ratio water to air was $[(\mu_{\text{en}}/\rho )w,\text{air}]5\;\text{cm}=1.032$ (TRS277, Table XIV), the perturbation factor was $p_{u}=1.03$ (TRS 277, Table XV), and the correction for spectral dependence was $k_{u}=1$.

The TLDs were irradiated in the same CT conditions as the ionization chamber and read with a Harshaw TLD Reader 4000 (Thermo Fisher Scientific Inc, Waltham, MA) associated to a GCA‐New v3.0 Ciemat software (Ciemat AMS Group, Madrid, Spain) using only the signal integration of the last two peaks.

A good predictor of the radiation risk for the patient is the effective dose. It can be obtained by taking the planar average equilibrium dose and multiplying by the scanning length and a conversion coefficient (“k”) which relates to the organs/tissues under consideration.^(2)^ These conversion coefficients are derived from mathematical phantoms using Monte Carlo modeling.^(12)^ The results obtained were compared with international references.^13^, ^14^, ^15^, ^16^

Free‐in‐air measurements were realized during acceptance tests and the equilibrium dose‐pitch product free‐in‐air was measured. This parameter can be checked in routine quality control and reveals the machine output constancy. For these measurements, the ionization chamber was attached on a plastic guide fixed to a laboratory stand in order to place the chamber far enough from the patient table to reduce scattered radiation. The chamber was aligned with the CT axis of rotation for “central” measures and then positioned at the same radial location that it had occupied for peripheral measurements in phantom. In our case with a pitch of 1, the equilibrium dose pitch product is equal to the equilibrium dose. The dose was evaluated in terms of air kerma. For each measure, the chamber starts entirely outside the irradiation beam, passes through the beam and finishes beyond the other side of the beam. For this reason, the scanning length would be superior to the chamber active length (1=23 mm)+ the total section width (nT=10 mm)+15 mm.^(8)^

## III. RESULTS & DISCUSSION

### A. Phantom and chamber positioning and dose repeatability

The water phantom central axis alignment with the CT rotation axis and the ionization chamber placement in the phantom were verified before each new measurement series in order to center the charge collection volume with the plane z=0 of the CT scanner. The setup position error was less than 1 mm.

The cable used was long enough to allow the placement of the electrometer outside the phantom scatter radiation field, and also to avoid induction of extra cameral currents in the electrometer itself. The stem effect or noise signal of the system was evaluated within 1%. The dose repeatability was evaluated 0.4%.

### B. Central (z=0)cumulative dose and its approach to equilibrium

#### B.1 Comparison of axial and helical scanning modes

**Figure 5:**
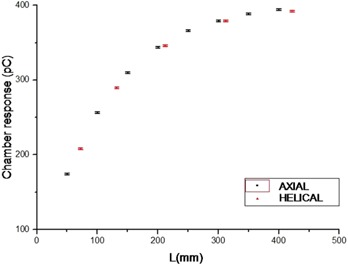
Comparison of doses delivered during both axial and helical scanning modes.

Figure 5 depicts the chamber response with the scanning length for both scanning modes. No significant differences were noticed if the two modes are comparable (adjacent slices for axial mode and pitch of 1 for helical mode) with an approach to equilibrium in the central hole close to 450 mm. For this reason, most of the following measurements were realized in axial mode, in order to simplify the scanning length determination.

### B.2 Approach to equilibrium of central‐ and peripheral‐axis central cumulative dose

**Figure 6:**
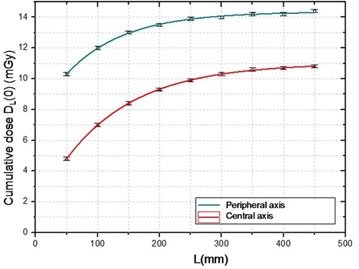
Approach to equilibrium of central and peripheral axes central cumulative doses.

The measurements for the reference set of operation in the central and peripheral holes are presented in Fig. 6 (dots). All the data (central‐ and peripheral‐axes central cumulative doses) were fitted by Eq. (1) with the Origin software (OriginLab, Northampton, MA), and are presented in Fig. 6 (lines).

The fitted parameters are summarized in Table 2. For central axis doses, where scatter contribution dominates the primary dose contribution, the α parameter is close to unity; $D_{\text{eq}}$ is lower for central axis (11.0±0.1  mGy) than for peripheral axis (14.4±0.1  mGy), and the length $L_{\text{eq}}$ for which the central cumulative dose is within 2% of $D_{\text{eq}}$ is higher for central axis (450 ± 1 mm) than for peripheral axis (380 ± 1 mm).

**Table 2:** Fitted parameters L_eq_, D_eq_ and a obtained with Origin software for both ionization chamber (IC) positions.

*IC POSITION*	*CENTRAL*	*PERIPHERAL*
$L_{\text{eq}}(\text{mm})$	450±1	380±1
$D_{\text{eq}}(\text{mGy})$	11.0±0.1	14.4±0.1
α	0.9	0.5

### C. Planar average equilibrium dose and integral dose

The planar average equilibrium dose $D_{\text{eq}}$ (Eq.2) and the integral dose $E_{\text{tot}}$ (Eq.3) obtained for the protocols used are summarized in Table 3. The planar average equilibrium dose was 12.4±0.1  mGy and the integral dose was evaluated at 395 ± 5 mJ for all protocols.

**Table 3:** Planar average equilibrium dose $D_{\text{eq}}$ and integral dose $E_{\text{TOT}}$ obtained for four sets of operating condition. Comparison between $D_{\text{eq}}$ and CTDI informed by the CT scanner.

*PROTOCOLS*	*PROTOCOL 1*	*PROTOCOL 2*	*PROTOCOL 3*	*PROTOCOL 4*
$D_{\text{eq},\text{central}}(\text{mGy})$	10.8±0.1	10.8±0.1	11.0±0.1	11.1±0.1
$D_{\text{eq},\text{peripheral}}(\text{mGy})$	13.8±0.1	13.8±0.1	14.0±0.1	13.7±0.1
$D_{\text{eq}}$ (mGy)	12.3±0.1	12.3±0.1	12.5±0.1	12.4±0.1
${\text{CTDI}}_{\text{vol}}$ (mGy)	9.1	9.3	9.3	9.3
Variation (%)	35.0%	32.4%	33.9%	33.3%
$E_{\text{TOT}}$ (mJ)	390±3	390±3	400±3	395±3

### D. Comparison with TLD measurements and CTDIvol informed by the CT scanner

#### D.1 TLD measurements

Following the TRS‐277 and using the signal integration of the last two peaks, the calibration factor for the TLD was 3.8E‐5 mGy/nC.

The comparison between the central and peripheral doses measured with the TLD and those measured with the ionization chamber is reported in Table 4. For all protocols, the planar average equilibrium dose $D_{\text{eq}}$ measured with TLD was evaluated at 12.3±0.2  mGy corresponding to a variation with the dose measured with ionization chamber lower than 2.0%. These measurements confirmed the correct setup and the methodology used during this work.

**Table 4:** TLD results and comparison with ionization chamber (IC) measurements.

*PROTOCOLS*	*PROTOCOL 1*	*PROTOCOL 2*	*PROTOCOL 3*	*PROTOCOL 4*
$D_{\text{eq},\text{central}}$ IC (mGy)	10.8±0.1	10.8±0.1	10.9±0.1	11.1±0.1
$D_{\text{eq},\text{central}}$ TLD(mGy)	10.4±0.2	10.7±0.2	10.9±0.2	11.0±0.2
Variation (%)	3.3%	0.7%	0.3%	0.6%
$D_{\text{eq},\text{periph}}$ IC (mGy)	13.8±0.1	13.8±0.1	14.0±0.1	13.7±0.1
$D_{\text{eq},\text{periph}}$_eq,periph_ TLD(mGy)	13.9±0.2	13.7±0.2	14.5±0.2	13.5±0.2
Variation (%)	0.7%	0.8%	‐4.0%	1.1%
$D_{\text{eq}}$ IC (mGy)	12.3±0.1	12.3±0.1	12.5±0.1	12.4±0.1
$D_{\text{eq}}$ TLD(mGy)	12.2±0.2	12.2±0.2	12.7±0.2	12.3±0.2
Variation (%)	1.0%	0.7%	‐1.9%	1.1%

#### D.2 Comparison with CTDIvol informed by the CT scanner

The ${\text{CTDI}}_{\text{vol}}$ indicated by the CT scanner for all protocols were between 32% and 35% inferior to the doses measured during this work. The results are presented in Table 3 and confirm that, in our case, the CTDI index is no longer adequate to determine the dose delivered during the scanner exam.

### E. Effective dose and comparison with international references

The average scanning lengths were respectively 350 ± 30 mm and 200 ± 50 mm for chest (Protocol 1) and prostate (Protocol 2) CT exams. The dose length products were 430 ± 37 (Protocol 1) and 246 ± 61 mGy/cm (Protocol 2). The k coefficients used were 0.014 mSv/(mGy.cm) for Protocol 1 and 0.015 mSv/(mGy.cm) for Protocol 2.^17^, ^18^ The effective doses calculated were 6.02±0.52  mSv for chest CT and 3.69±0.75  for prostate CT scan. The published values are between 5 and 8 mSv for chest localization and between 6 and 9 mSv for pelvis CT.^13^, ^14^, ^15^, ^16^ The effective doses calculated during this work are below the international published values, which reveals good practice.

### F. Free‐in‐air equilibrium dose

In our case the scanning length used was 50 mm. For this scanning length of exploration, the equilibrium dose‐pitch product $D_{\text{eq},\mathrm{air}}$ free‐in‐air for all sets of operating conditions and was 27.5±0.2  mGy per 100 mAs for the chamber aligned along the axis of rotation of the CT scanner, and 14.2±0.2  mGy per 100 mAs for the chamber placed at the same radial location as the location used with the water phantom. It appears very important to establish the free‐in‐air equilibrium dose during acceptance tests in order to verify the machine output during routine quality controls.

## IV. CONCLUSIONS

As predicted by the AAPM TG111 Report, this work showed the limitation of the CTDI and, more particularly, of the index ${\text{CTDI}}_{100}$. This index is a simple standardized measure of the dose output of the CT scanner and can therefore be used during quality assurance or to compare different scan techniques. Regarding the doses really delivered to the patient during CT exams, it revealed a systematic underestimation (30%–35%). The doses informed by the CT scanner at the end of the exams are still based on the CTDI paradigm. For new generations of CT systems with wider longitudinal detector size or cone‐beam technology, it is therefore important to evaluate CT radiation dose with new methodology. The AAPM report proposed new metrics easy to follow and to implement using a small active volume ionization chamber commonly used in radiotherapy services calibrated in the CT beam quality range. The TLD measurements validated the correct measurements setup and the AAPM methodology. Moreover, the effective dose mirrors the radiation risk and, therefore, it is very important to determine it. The CT scanner output can be easily verified with free‐in‐air measures.
